# An embedded and embodied neuroscience of interpersonal synchrony

**DOI:** 10.1093/oons/kvag017

**Published:** 2026-10-27

**Authors:** Manuel Varlet, Michael J Richardson

**Affiliations:** The MARCS Institute for Brain, Behaviour and Development, Western Sydney University, Locked Bag 1797, Penrith, NSW 2751, Australia; School of Psychology, Western Sydney University, Locked Bag 1797, Penrith, NSW 2751, Australia; School of Psychological Sciences, Macquarie University, 16 University Avenue, Sydney, NSW 2109, Australia; Performance and Expertise Research Centre, Macquarie University, 16 University Avenue, Sydney, NSW 2109, Australia

**Keywords:** interpersonal synchrony, dynamical systems, self-organisation, multiscale synergies, embodiment, collective variables

## Abstract

Interpersonal synchrony provides a powerful framework for understanding how coordinated behaviour emerges across interacting individuals. While neuroscientific advances have extended this work to the neural level, they risk reducing synchrony to isolated mechanisms without an appropriate conceptual framework. We argue for an embedded and embodied approach in which neural, physiological, and behavioural processes are treated as co-constitutive components of coupled systems. Within this perspective, neural activity both constrains and is constrained by the dynamics of interaction. We highlight key challenges, including isolating genuine neural signals, developing multi-level dynamical models, and identifying collective variables linking scales. We also set out criteria for identifying such variables and predictions by which the framework can be tested. Rather than abandoning mechanism, this approach redefines it as multilevel and dynamical, providing a principled basis for a more integrative science of interpersonal synchrony.

## Introduction

Interpersonal synchrony has attracted considerable attention across disciplines, including psychology, psychiatry, robotics, music, language, sports, and neuroscience (Keller et al. 2014, Navare et al. 2024, Schmidt and Richardson 2008, Sebanz et al. 2006). Synchronised behaviour supports communication, coordination, and social connectedness across the lifespan, from early development to older age (Cirelli et al. 2014, Marsh et al. 2009a, Miles et al. 2023, Richardson et al. 2007a), and its disruption is increasingly recognised as an early marker of social and neurodevelopmental disorders (Fitzpatrick et al. 2016, Kupper et al. 2015, Macpherson and Miles 2023, Moreau et al. 2024, Pan et al. 2023, Raffard et al. 2015, Varlet et al. 2012a, Varlet et al. 2014a). More broadly, synchrony underpins collective achievements from joint musical performance to skilled team sports, shaping the organisation of human behaviour across social systems (D’Ausilio et al. 2015, Duarte et al. 2013, Konvalinka et al. 2011, MacRitchie et al. 2017, Wiltermuth and Heath 2009).

For decades, research on interpersonal synchrony was grounded in behavioural methods, in part because neuroscientific tools capable of capturing the intrinsically dynamic, interactive, and multi-person nature of social coordination were limited. This work nonetheless produced substantial advances, supported by extensive theoretical debate spanning computational and dynamical systems perspectives (Schmidt and Richardson 2008, Sebanz et al. 2006, Coey et al. 2012, Marsh et al. 2009b, Sebanz and Knoblich 2009, Sebanz and Knoblich 2021). Over the past fifteen years, the field has increasingly turned toward neuroscientific approaches, driven by advances in multi-brain neuroimaging and stimulation (Dikker et al. 2017, Dumas et al. 2010, Montague et al. 2002, Novembre and Iannetti 2021a, Schilbach and Redcay 2025). These developments hold promise, but have often proceeded with limited integration of prior behavioural research and theory, and with a focus on the brain that privileges internalist interpretations positioning neural processes as the primary drivers of synchrony. Current accounts thus borrow analytical tools from the dynamical systems approach without incorporating its broader theoretical principles.

Here we argue for a renewed integration of dynamical systems principles into the study of interpersonal synchrony, bridging its behavioural, theoretical, and neuroscientific strands. Decades of behavioural work have established synchrony as a lawful, self-organising phenomenon emerging from coupled social systems rather than solely from processes internal to each individual. Our aim here is to situate brain processes within that broader interactive dynamic.

In arguing this we build on a broader movement to place behaviour at the centre of neuroscientific explanation. Krakauer and colleagues (Krakauer et al. 2017) have argued that detailed behavioural characterisation must precede and constrain the search for neural mechanism. We share that commitment but extend it. For interpersonal synchrony the system of interest is not the individual organism but the dyad and the medium coupling it, so the variables carrying the explanation are dyad-level and have no single-brain counterpart.

We use interpersonal synchrony as the field’s conventional umbrella term, but the framework concerns coordination dynamics more generally. Synchrony proper, the convergence of coupled dynamics on a stable phase relation, is one region of a space that also includes anti-phase patterns, turn-taking, complementary action, leader–follower asymmetry, and metastable regimes in which a dyad visits coordination tendencies without settling into them (Tognoli and Kelso 2014). Effective interaction does not require maximal synchrony.

## Interpersonal synchrony as a dynamical system

Interpersonal synchrony is best understood as a dynamical systems phenomenon: it arises in ensembles of interacting components whose behaviour evolves over time through lawful relations (Schmidt and Richardson 2008, Marsh et al. 2009a, Kelso 1995, Pikovsky et al. 2001, Schmidt et al. 1990, Schmidt and O’Brien 1997, Strogatz 2003). From this perspective, synchrony is not defined by a central controller, a pre-specified program, or the alignment of independently specified internal representations. Coordinated patterns emerge instead from the continuous coupling between systems, in which each component both constrains and is constrained by the others. Our target is not computation as such, since the coupled-oscillator models discussed below are themselves computational. We reject accounts in which the causal explanations are located primarily within individual brains and interaction is a downstream consequence.

The order is therefore not driven but self-organised, arising spontaneously through interaction without an external coordinator or hierarchical control. Huygens observed as much in the 17th century, when two pendulum clocks on a common support gradually drew their motions into synchrony (Huygens 1897, Pena Ramirez et al. 2016); the same principle is seen when metronomes on a freely moving platform exchange energy through the shared base until their rhythms align (Pantaleone 2002). Such mechanical demonstrations illustrate self-organisation as a general principle, even though the coupling between interacting people is informational rather than mechanical, a distinction we develop below. When coupling is sufficient, stable coordination emerges as an attractor of the dynamics: patterns such as phase locking or rhythmic alignment are not imposed but arise as solutions of the system’s organisation, reflecting the tendency of coupled systems to reduce their degrees of freedom into coherent collective states.

Work on coupled oscillators shows synchronisation to be ubiquitous, observed across mechanical oscillators, chemical reactions, and biological rhythms (Pikovsky et al. 2001, Strogatz 2003), from fireflies that flash in unison through local visual coupling (Buck 1988, Mirollo and Strogatz 1990) to neuronal populations coupled through excitatory and inhibitory synaptic interaction (Belykh and Shilnikov 2008, Buzsáki 2006). Across these systems, complex collective behaviour can often be captured by low-dimensional collective variables, such as relative phase, that describe coordination between components and govern its stability, transitions, and variability (Kelso 1995, Haken 1977).

This framework shifts the explanatory focus from isolated components to the organisation of the system as a whole. The relevant level of description becomes the interaction itself, where coordination is an emergent property of coupled dynamics rather than the output of independent processes (Schmidt and Richardson 2008, Kelso 1995, Schmidt et al. 1990, Strogatz 2003). Causality is therefore distributed and reciprocal, unfolding through continuous mutual influence; what appears as coordinated behaviour is the manifestation of system-level organisation rather than the expression of hidden internal states.

Coupling, self-organisation, low-dimensional collective variables, and universality across systems thus form a general framework for understanding synchrony as the emergent outcome of interacting systems governed by shared dynamical laws.

## Self-organising synchrony: Behavioural evidence

A large body of behavioural evidence shows that synchrony emerges as a robust, self-organising phenomenon across experimental and ecological contexts (Schmidt and Richardson 2008, Marsh et al. 2009a, Coey et al. 2012, Kelso 1995, Riley et al. 2011). In both dyadic and group settings, coordinated patterns arise spontaneously from interaction, without explicit instruction, central control, or predefined roles, in tasks ranging from simple laboratory paradigms to complex whole-body activities (Miles et al. 2023, Schmidt et al. 1990, Coey et al. 2011, Miyata et al. 2017, Néda et al. 2000, Richardson et al. 2007b).

The foundational studies established these principles with tightly controlled rhythmic tasks. When pairs were asked to swing their lower legs in-phase (0°) or anti-phase (180°), increasing movement frequency destabilised the anti-phase pattern and produced spontaneous transitions to in-phase coordination, with the critical fluctuations and hysteresis that mark a dynamical phase transition (Schmidt et al. 1990). Coordination emerged even without instruction: when participants merely oscillated hand-held pendulums while observing one another, their movements became more coherent and converged toward relative phases near 0° and 180° (Schmidt and O’Brien 1997, Richardson et al. 2005). Interpersonal coordination thus arises from lawful coupling between oscillatory systems and is governed by collective variables such as relative phase.

These regularities extend well beyond the laboratory. Audience applause can synchronise spontaneously as individuals adjust to those around them, with no conductor or imposed pacing (Néda et al. 2000), and comparable unintentional synchrony appears in rocking chairs (Richardson et al. 2007b, Richardson et al. 2012), postural sway (Miyata et al. 2021, Varlet et al. 2011, Varlet et al. 2014b), and locomotion (Felsberg and Rhea 2021, van Ulzen et al. 2008, Varlet and Richardson 2015). Across contexts, the strength and stability of coordination track the availability and quality of perceptual coupling (Richardson et al. 2007b, Richardson et al. 2005, Varlet et al. 2012b, Varlet et al. 2015).

Coupled-oscillator models capture these regularities across scales. For dyads, the Haken–Kelso–Bunz model formalises synchrony as coupled nonlinear oscillators, deriving in-phase and anti-phase patterns as stable solutions whose relative stability depends on system parameters (Kelso 1995, Fuchs et al. 1996, Haken et al. 1985); reducing behaviour to a single collective variable (relative phase) yields a parsimonious yet nonlinear account. Kuramoto-inspired extensions scale the same logic to groups, where many oscillators interact through distributed coupling to produce partial or global synchrony (Acebrón et al. 2005, Alderisio et al. 2017, Bardy et al. 2020, Frank and Richardson 2010). Asymmetry is native to these models rather than an exception to them: differences in the partners’ preferred frequencies (detuning) produce systematic phase lead and lag, and asymmetric coupling produces leader–follower structure (Varlet et al. 2014a, Fuchs et al. 1996, Varlet et al. 2020). This is the regime that characterises clinician–patient, caregiver–infant, and instructional dyads, and in which difficulty leading is itself diagnostic.

Interpersonal synchrony is therefore a lawful and scalable phenomenon: across tasks, modalities, and levels of complexity, coordinated behaviour can be understood as the emergent outcome of coupled oscillatory dynamics. The question is how neuroscience fits within that account, to which we now turn.

## Current neuroscientific approaches to interpersonal synchrony

Neuroscientific approaches to interpersonal synchrony have expanded rapidly, driven by multi-person recording techniques (Dumas et al. 2010, Schilbach and Redcay 2025, Babiloni and Astolfi 2014, Czeszumski et al. 2020, Zamm et al. 2024). Hyperscanning methods, including multi-participant EEG, MEG, fNIRS, and fMRI (Dumas et al. 2010, Montague et al. 2002, Czeszumski et al. 2020, Cui et al. 2012, Lindenberger et al. 2009, Moerel et al. 2025), now measure neural activity across interacting individuals in real time. Foundational studies demonstrated interbrain synchronisation in both visual and auditory interaction: dual-EEG during spontaneous imitation of hand movements revealed alpha–mu synchronisation across right centroparietal regions (Dumas et al. 2010), and guitarists in duet showed phase synchronisation that covaried with the temporal demands of coordinated performance (Lindenberger et al. 2009). Subsequent work has identified interbrain synchronisation across many frequencies, regions, and contexts, typically quantified through phase alignment, coherence, or cross-correlation (Schilbach and Redcay 2025, Babiloni and Astolfi 2014, Czeszumski et al. 2020, Zamm et al. 2024).

A persistent challenge has accompanied this growth: determining whether observed interbrain synchrony reflects genuine neural coupling or an epiphenomenal by-product of shared behaviour (Novembre and Iannetti 2021a, Schilbach and Redcay 2025, Burgess 2013, Hamilton 2021, Hari et al. 2013, Holroyd 2022, Varlet and Grootswagers 2024, Hamilton and Konvalinka 2026). Behavioural synchrony can be reflected in neural signals through several confounds, including motor artefacts (e.g. movements of the body, face, or eyes contaminating EEG) and parallel but independent responses to shared sensory input and task constraints. In such cases neural alignment does not need to index interpersonal coupling, but only concurrent engagement with the same environment.

In response, the field has sought causal relevance rather than correlation (Novembre and Iannetti 2021a, Moreau and Dumas 2021, Novembre and Iannetti 2021b), much of the effort turning on directionality: whether synchronised neural oscillations drive coordinated behaviour or instead follow from it. Because neural oscillations are widely treated as internal timing devices (Fries 2015, Varela et al. 2001), their cross-individual alignment is hypothesised to shape interpersonal behaviour.

Recent work has moved from observing interbrain synchrony to probing its causal role directly (Novembre and Iannetti 2021a, Moreau and Dumas 2021, Leiva-Cisterna et al. 2025, Novembre et al. 2017). One line entrains oscillations across individuals: with transcranial alternating current stimulation (tACS), Novembre et al. (2017) found that phase-synchronised dual-brain stimulation at 20 Hz enhanced behavioural synchrony in joint finger-tapping. A complementary line manipulates shared sensory input; Leiva-Cisterna et al. (2025) used rhythmic visual flicker to induce shared oscillatory dynamics and reported enhanced dyadic cooperation. Such designs are argued to adjudicate between mechanistic and epiphenomenal accounts.

This search for mechanism has, however, pushed the field toward a brain-centred account, in which causal explanations are sought primarily within individual brains, while the body and environment are reduced to peripheral roles as inputs and outputs. Even when it borrows dynamical systems tools (such as phase-synchrony measures intended to capture self-organising, intermittent attraction in complex systems), it tends to reduce the findings to processes within individual brains, framing coordination as the alignment of internally generated neural states rather than as an emergent property of coupled systems. A dynamical measure does not, by itself, constitute a dynamical explanation. The vocabulary can be imported while the underlying commitment to brain-internal causation is left intact.

This perspective is generative but limited. By treating the brain as the primary locus of causation, it underweights how brains both shape and are shaped by their social and ecological context (Schmidt and Richardson 2008, Coey et al. 2012, Marsh et al. 2009b, Richardson and Chemero 2014). That synchrony appears concurrently at behavioural, physiological, and neural scales does not by itself establish irreducibility; but the consistent cross-scale coupling, together with the confounds above, makes a brain-only account unlikely to be sufficient (Konvalinka et al. 2011, Chidichimo et al. 2026, Mayo and Gordon 2020, Moffat et al. 2026). Advancing the field therefore calls for more than moving beyond correlation; it calls for a shift in explanatory focus, treating neural activity as one component of a broader, dynamically coupled system in which coordination arises through self-organising synergies across levels (Kelso 1995, Riley et al. 2011, Richardson and Chemero 2014, Chidichimo et al. 2026).

## Rethinking the neuroscience of interpersonal synchrony

Meeting these challenges requires a decisive shift toward an interaction-centred neuroscience of interpersonal synchrony (Dumas 2011, Redcay and Schilbach 2019, Schilbach et al. 2013). The aim is not to explain synchrony at the level of the brain alone, but to identify how neural activity participates in, and is shaped by, the coordinated dynamics of the interacting system as a whole (Coey et al. 2012, Marsh et al. 2009b, Zamm et al. 2024, Chidichimo et al. 2026). By interaction-centred we mean that the interaction, not the individual organism, is the primary unit of analysis. By embodied we mean that the body’s dynamics are part of the coordinating system rather than a peripheral implementation of it. By embedded we mean that the shared environment through which people perceive one another is a constituent of that system. This approach builds on a longer tradition treating interaction as a unit of analysis, from work on a socially situated nervous system (Coey et al. 2012), social coordination dynamics (Kelso et al. 2013, Dumas et al. 2014), and informational accounts of coordination (Chidichimo et al. 2026) to broader enactive and predictive approaches to social cognition (De Jaegher and Di Paolo 2007, Friston and Frith 2015, Monnier et al. 2025). It differs in treating the informational coupling between people as constitutive of a single embodied system rather than a quantity measured between separate brains, and in making this testable through explicit criteria for collective variables, concrete predictions, and conditions under which the framework fails.

Within such a framework, neuroscience has the potential to clarify the nature of interpersonal coupling (Coey et al. 2012, Zamm et al. 2024, Dumas and Fairhurst 2021, Wolff and Dumas 2026). Unlike within-brain coupling, interpersonal coupling is fundamentally informational (Schmidt and Richardson 2008, Kelso 1995, Miyata et al. 2017, Richardson et al. 2007b): synchrony emerges through the continuous exchange of perceptual information, particularly the visual and auditory signals generated by behaviour. From this perspective, neurophysiological methods are well suited to investigating how sensory information is selectively processed and transformed into the interaction-relevant information that supports synchrony (Paas et al. 2021, Painter et al. 2021, Varlet et al. 2025, Zhou et al. 2026).

A central methodological challenge follows: isolating genuine neural activity, particularly sensory processing related to interpersonal coupling, within neuroimaging signals recorded during movement (Burgess 2013, Hamilton 2021, Holroyd 2022). Measurement artefacts (i.e. ocular, muscular, and motion-induced contamination) must be removed, but movement-related neural activity remains of particular interest. Motor preparation, execution, and sensorimotor prediction are constitutive of the coordinating system on an embodied account, and regressing them out discards part of the phenomenon. Rather, the challenge is to dissociate neural activity related to visual and/or auditory coupling from motor-related activity. The E-music box decouples movement from acoustic outcome, so that identical auditory results can be produced by different movements and different results by the same movement. This separates sensory from motor contributions within a genuinely interactive task, rather than by stripping the interaction away (Novembre et al. 2015); using it, Zhou et al. (2026) found that within-brain attentional engagement with shared auditory information predicted interpersonal synchrony and joint agency more strongly than interbrain synchrony did. A brain-to-brain framing struggles to accommodate that result, whereas it follows naturally if coordination is carried by the information passing between people. EEG frequency-tagging achieves a comparable separation: sensory information related to self- and other-generated movement is tagged at distinct frequencies, allowing these two sources of information to be separated from one another and from participant’s own motor activity in neural recordings (Varlet et al. 2020, Painter et al. 2021, Bánki et al. 2024). In a visual mirror-game task, Varlet et al. (2020) showed that neural tracking and integration of information related to self- and other-generated movement predicted synchrony performance. The same logic extends to audition. With each partner’s sound tagged at a distinct frequency, stronger neural tracking of self-generated sound and self–other separation (indexed by intermodulation responses) were associated with better synchrony (Varlet et al. 2026). In short, standard hyperscanning asks whether two brains covary. These paradigms ask what information passes between two people and how each nervous system takes it up.

More generally, progress in this area will require integrating neural, behavioural, physiological, and environmental dynamics within a single dynamical framework (Coey et al. 2012, Kelso 1995, Richardson and Chemero 2014, Chidichimo et al. 2026, Mayo and Gordon 2020). Interpersonal synchrony emerges through nonlinear interactions unfolding across multiple spatial and temporal scales, with reciprocal influences operating both within and across levels. These scales are separated by orders of magnitude (neural dynamics on the order of milliseconds, behavioural coordination over hundreds of milliseconds to seconds, cardiac and respiratory dynamics over seconds to minutes). How they are to be integrated remains an open problem, not a solved one (Fig. 1).

**Figure 1:**
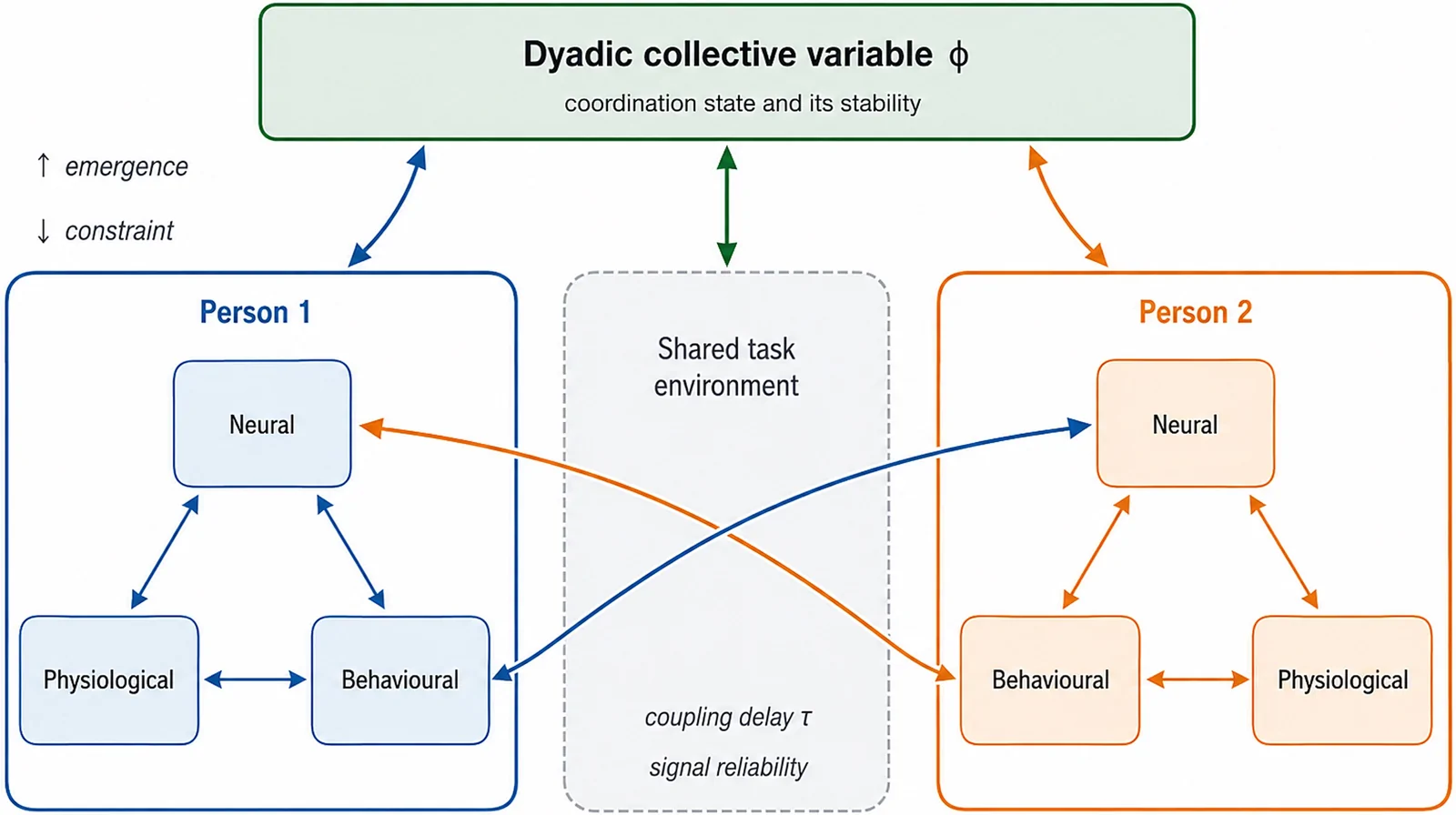
Interpersonal synchrony as a multilevel dynamical system. Within each person, neural, physiological, and behavioural processes are reciprocally coupled at every pair. The two people are in turn coupled to one another informationally: Each person’s behaviour structures the shared task environment, which carries its own coupling delay and signal reliability, and from which the other perceives. Coordination is therefore organised by a dyad-level collective variable that emerges from the coupled system and constrains it in return, acting on the components and on the interaction rather than being located within either brain.

Methodologically, this reorientation means moving beyond static or pairwise measures toward methods that capture time-varying, multilevel dynamics in naturalistic settings. Such approaches should identify collective variables that span organisational scales and whose patterning, stability, and transitions can be modelled within low-dimensional dynamical systems. What qualifies a variable as collective, as opposed to a convenient multimodal summary, is not the number of levels it draws on but the work it does. First, it is defined over the relation between components and is not recoverable from individual measures taken separately (relative phase qualifies, whereas the mean of two movement amplitudes does not). Second, it is low-dimensional, compressing the component description rather than accumulating it. Third, it behaves as an order parameter, showing differential stability across coordination states, critical fluctuations and hysteresis near transitions, and the enslavement of component dynamics. Fourth, its dynamics are constrained by, and in turn constrain, processes at neural, bodily, and environmental levels (Fig. 1). A multimodal summary describes the system, whereas a collective variable governs it. The third criterion is the empirical test that separates them. In the mirror game, relative phase between partners’ movements is the candidate collective variable; movement kinematics, cardiac, and respiratory phase are component variables; and frequency-tagged neural responses index the informational coupling, whose delay and signal reliability are environmental variables.

Nonlinear dynamical systems methods (e.g. phase-based modelling, dynamic network approaches, and recurrence and cross-recurrence analyses) are suited to characterising reciprocal dependencies, metastable coordination, and interaction dynamics across temporal and organisational scales (Tognoli and Kelso 2014, Bassett and Sporns 2017, Coco and Dale 2014, Marwan et al. 2007, Richardson et al. 2014). Complementing these approaches, information-theoretic measures relate variables that are not otherwise commensurable: mutual information quantifies dependence without assuming linearity, transfer entropy adds directionality, and partial information decomposition separates unique, redundant, and synergistic contributions (Chidichimo et al. 2026). Synergy is of particular interest, since information about the coordination state carried jointly by neural, bodily, and environmental variables but by none alone is the formal expression of the claim made here. These measures are not, however, tests of causation. Transfer entropy is sensitive to common drivers, unobserved variables, lag and embedding choice, and non-stationarity, and a dyad sharing an environment is exactly where common input is most likely to inflate it. They are best treated as hypothesis-generating, with causal claims resting on perturbation.

These commitments are testable. A multilevel account predicts that models including behavioural, physiological, and environmental variables will outperform neural-only models in predicting coordination states and their transitions, and that dyad-level collective variables will explain transitions beyond individual-level measures and their interactions. It further predicts that perturbing the coupling medium (its delay, reliability, or availability) will reorganise neural and behavioural dynamics together, and that information about the coordination state will prove synergistic across levels. The framework’s added value is not established if neural-only models match multilevel ones, dyad-level variables add nothing beyond individual measures, perturbing the coupling medium leaves neural dynamics unchanged, and no synergistic information is found across levels.

Advancing the neuroscience of interpersonal synchrony thus means moving the explanatory focus from localisation to organisation: from where synchrony occurs to how coordinated dynamics emerge, stabilise, and transform. Mechanism is not abandoned but redefined: multilevel and dynamical, with neural processes organised in relation to interpersonal synergies across scales.

## Conclusion

Interpersonal synchrony offers a unique window into the organisation of behaviour across interacting systems. Decades of behavioural research establish it as a lawful, self-organising phenomenon grounded in dynamical principles that transcend particular tasks or contexts, and recent neuroscience has extended this to the level of neural activity. Realising that potential, however, requires a conceptual realignment: without the theoretical foundations of dynamical systems, neuroscientific approaches risk reproducing the very limitations that behavioural work overcame. A mechanistic understanding of synchrony will not come from reducing it to internal processes, but from a multilevel, dynamical account of the system as a whole, within which neural activity both constrains and is constrained by ongoing embodied interaction. That is the integrative and embedded framework a dynamical systems perspective makes possible, and the programme we hope this Special Issue will advance.

## Data Availability

No new data were generated or analysed in support of this article.
